# Divergent Synthesis of Novel Cylindrocyclophanes that Inhibit Methicillin‐Resistant *Staphylococcus aureus* (MRSA)

**DOI:** 10.1002/cmdc.202000179

**Published:** 2020-06-12

**Authors:** Julien J. Freudenreich, Sean Bartlett, Naomi S. Robertson, Sarah L. Kidd, Suzie Forrest, Hannah F. Sore, Warren R. J. D. Galloway, Martin Welch, David R. Spring

**Affiliations:** ^1^ Department of Chemistry University of Cambridge Lensfield Road Cambridge CB2 1EW UK; ^2^ Department of Biochemistry University of Cambridge Downing Site Cambridge CB2 1QW UK

**Keywords:** cross metathesis, cylindrocyclophane, macrocycles, ring-closing metathesis

## Abstract

The cylindrocyclophanes are a family of macrocyclic natural products reported to exhibit antibacterial activity. Little is known about the structural basis of this activity due to the challenges associated with their synthesis or isolation. We hypothesised that structural modification of the cylindrocyclophane scaffold could streamline their synthesis without significant loss of activity. Herein, we report a divergent synthesis of the cylindrocyclophane core enabling access to symmetrical macrocycles by means of a catalytic, domino cross‐metathesis‐ring‐closing metathesis cascade, followed by late‐stage diversification. Phenotypic screening identified several novel inhibitors of methicillin‐resistant *Staphylococcus aureus*. The most potent inhibitor has a unique tetrabrominated [7,7]paracyclophane core with no known counterpart in nature. Together these illustrate the potential of divergent synthesis using catalysis and unbiased screening methods in modern antibacterial discovery.

## Graphical abstract

**Something new**: The synthesis of novel cylindrocyclophanes varying in ring size, oxidation level and functionalisation is described. Key steps include a catalytic, domino cross‐metathesis‐ring‐closing metathesis cascade, and late‐stage diversification of the resulting resorcinol core. Antibacterial evaluation showed that the modifications are tolerated and we used this approach to identify several novel inhibitors of *S. aureus* and MRSA.

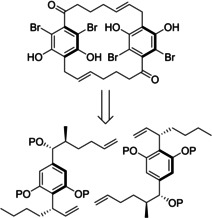

## Introduction

*Staphylococcus aureus* is a serious cause of community‐ and healthcare‐associated infection worldwide.^1^ A particular health burden is the treatment of methicillin‐resistant *S. aureus* (MRSA) infection, which is associated with a significant increase in mortality and long‐term patient care.^2^ As such, the World Health Organization has recently designated MRSA as a high‐priority pathogen for focused antibacterial research and development.^3^

New antibiotics are needed just to keep up with the spread of resistance, but this need is not being met by the development pipeline.^4^ For decades, pharmaceutical companies have struggled with the complexities of bringing novel antibiotics to market.^5^, ^6^ Accordingly, most antibiotics available today are derivatives of older antibiotics that have since been phased out. This commonality limits the lifespan of new treatments before cross‐resistance renders them ineffective.^7^

**Figure 1:**
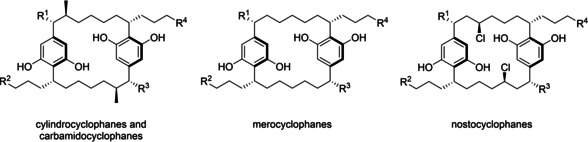
Structural features of [7.7]paracyclophane natural products. All share a dimeric alkylresorcinol motif but differ in substitution pattern. R^1^–R^4^ represent side chain substituents.

In an attempt to break this deadlock, recent years have seen growing interest in the exploration of new antibacterial scaffolds and targets in screening.^8^ In particular, we and others have sought to make use of divergent synthesis to identify novel antibacterial leads for drug development.^9^, ^10^, ^11^ The cylindrocyclophanes are a family of macrocyclic natural products isolated from marine and terrestrial cyanobacteria.^12^, ^13^, ^14^ They are structurally related to the corresponding carbamido‐, nosto‐ and merocyclophanes, which share a common [7.7]paracyclophane backbone but vary in α‐, β‐ and peripheral substitution patterns and oxidation level (Figure 1).^15^, ^16^, ^17^, ^18^, ^19^, ^20^ For an excellent review on alkylresorcinols such as cylindrocyclophanes, see Martins et al.^21^

The biochemical and chemical synthesis of cyclophane natural products has interested and occupied chemists for decades.^22^, ^23^, ^24^, ^25^, ^26^, ^27^, ^28^, ^29^, ^30^, ^31^ Several reports describe the antibacterial activities of related carbamidocyclophane natural products; however, the cylindrocyclophanes have been subject to rather less attention in this regard. To our knowledge, all studies to date describing the antibacterial evaluation of the cylindrocyclophane family are restricted to naturally occurring [7.7]paracyclophanes of which 16 members have been identified.^32^, ^33^ This limits the chemical diversity and hence scope of any such investigation, meaning that little is known about the structure–activity relationships of these compounds or their derivatives. The cylindrofridins (linear congeners of the cylindrocyclophanes) display reduced activity against MRSA and *Streptococcus pneumoniae*, thus suggesting that cyclisation augments the antibacterial activity of this scaffold.^32^ The cylindrocyclophane α‐OH motif is not required for activity against *Mycobacterium tuberculosis*, although α‐acetylated cylindrocyclophanes display reduced activity against MRSA.^33^

These observations prompted us to question which structural motifs might be responsible for the antibacterial activity of the cylindrocyclophanes. We thought it possible that we could design cylindrocyclophane analogues with streamlined syntheses that retain the antibacterial activity of the parent scaffold. If so, this would significantly reduce the effort needed to synthesise and study this family of compounds. As such, we sought to develop of a chemical synthesis of cylindrocyclophane scaffolds to enable the exploration of the cylindrocyclophanes as novel antibacterials. Herein, we report the divergent synthesis of a collection of novel cylindrocyclophanes varying in both ring architecture and functionalisation around the core. Phenotypic screening was performed using the library of novel cylindrocyclophane analogues against a panel of common clinical pathogens. The results of the phenotypic screening and implications of these findings for the discovery of novel antibacterials using divergent synthesis are discussed.

## Results

**Scheme 1:**
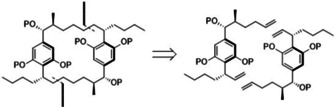
Disconnection of the cylindrocyclophane core into symmetrical monomers. Dashed lines indicate the intended location of bond disconnection. P=protecting group.

We sought an efficient route towards a simplified cylindrocyclophane core. We envisaged a key disconnection of the cylindrocyclophane scaffold into symmetrical monomers. We hoped to make use of a domino cross‐metathesis‐ring‐closing metathesis cascade as the pivotal ring‐forming step based on recent syntheses that have employed similar head‐to‐tail cyclodimersations to good effect.^26^, ^27^, ^28^, ^29^, ^30^, ^34^ From here, we felt that this common intermediate would be accessible from commercial materials in just a few steps (Scheme 1). This head‐to‐tail cyclodimerisation would enable the study of a homologous series of symmetrical [*m.n*]cylindrocyclophanes through variation of the chain length installed during synthesis. We hoped to use this route to investigate the role of the resorcinol core by testing protected derivatives, as well as the role of the substituent in the α‐position, further unsaturation and other late‐stage modifications of the parent scaffold.

**Scheme 2:**
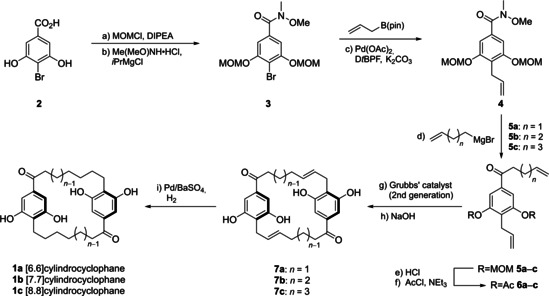
Synthesis of cylindrocyclophanes **1 a**–**1 c**. a) chloromethyl methyl ether (3.3 equiv), *N*,*N*‐diisopropylethylamine/CH_2_Cl_2_ (1 : 1), 0 °C to rt, 16 h, quant.; b) Me(MeO)NH⋅HCl (1.6 equiv), *i*PrMgCl (3.2 equiv), THF, −10 °C, 30 min, 75 %; c) Pd(OAc)_2_ (3 mol %), 1,1′‐bis(di‐*tert*‐butylphosphino)ferrocene (3.6 mol %), K_2_CO_3_ (3 equiv), allylboronic acid pinacol ester (2.5 equiv), THF, reflux, overnight, 78 %; d) alkenyl magnesium bromide (2 equiv), THF, 0 °C to rt, 2 h, 72–89 %; e) HCl/MeOH (1 : 2), 60 °C, 1–3 h, quant.; f) NEt_3_ (4.4 equiv), AcCl (4.4 equiv), CH_2_Cl_2_, 0 °C to rt, overnight, 66–87 %; g) Grubbs’ 2nd‐generation catalyst (5 mol %), CH_2_Cl_2_, reflux, 20 h, 4–61 %; h) NaOH (12 equiv), MeOH/CH_2_Cl_2_/H_2_O (4 : 1 : 1), rt, 1 h, 84–89 %; i) H_2_ (1 atm), Pd/BaSO_4_ (10 wt %), acetone, rt, overnight, 35–60 %.

**Scheme 3:**
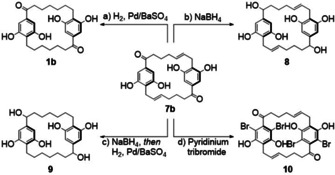
Late‐stage diversification of [7.7]cylindrocyclophane **1 b**. a) H_2_ (1 atm), Pd/BaSO_4_ (10 wt %), acetone, rt, overnight, 35 %; b) NaBH_4_ (4.8 equiv), MeOH, rt, 30 min, 55 %; c) NaBH_4_ (4.8 equiv), MeOH, rt, 30 min, then Pd/BaSO_4_ (10 wt %), acetone, rt, overnight, 68 % for second step; d) pyridinium tribromide (2.4 equiv), EtOH, rt, overnight, 23 %.

Gratifyingly, the synthesis of cylindrocyclophanes **1 a**–**c** could be achieved effectively in this way (Scheme 2). Manipulation of acid **2** provided the Suzuki substrate **3**, which was coupled with the allylic boronate ester to provide Weinreb amide **4** in good yield to serve as the branching point in our synthesis. Reaction with the *n*‐alkenyl Grignards yielded compounds **5 a**–**c**, which constitute a homologous series of acyclic precursors varying in alkenyl chain length. Subsequent deprotection and acetylation of **5 a**–**c** yielded the requisite precursors **6 a**–**c** for our domino cross‐metathesis‐ring‐closing metathesis end‐game (Scheme 3).

Treatment of **6 a**–**c** with Grubbs’ catalyst, followed by hydrolysis of the acetyl protecting group promoted the desired regioselective cyclodimerisation to construct the [*m.n*]paracyclophane scaffolds **7 a**–**c**. Predominant head‐to‐tail cyclodimerisation was confirmed by single‐crystal X‐ray diffraction of **7 b**, and for all other analogues by analogy. During reaction we saw trace trimerisation of **6 a** and **6 c**. In both cases regioselectivity was poor; symmetric and asymmetric trimers **11 a** and **11 c** were formed and purified in almost equal amounts. Finally, olefin reduction was achieved using hydrogen over palladium on barium sulfate to conclude our synthesis of the desired cyclindrocyclophane analogues **1 a**–**c**. Using this route we were able to prepare more than one gram of [7.7]cylindrocyclophane **1 b** for study in eight steps and 25 % overall yield (cf 11–16 steps, 8–22 % yield for the natural products).

We were able to build on this synthesis by diversifying the unsaturated [7.7]paracyclophane intermediate **7 b** at this stage. As mentioned, conversion to **1 b** was achieved using hydrogen and palladium over barium sulfate. In addition, reduction of **7 b** to 8 and doubly reduced cylindrocyclophane **9** completed this set of structural analogues, enabling us to investigate the effect of sequential reductions upon the antibacterial activity of this scaffold. Finally, based on a series of isolated natural cyclophanes with some uncommon substituents we also sought to investigate a particularly unusual modification to the cylindrocyclophane core to see if bromination had an effect of antibacterial activity. Previous work has identified a family of brominated cylindrocyclophanes isolated from Nostoc when cultured under particular conditions.^14^ Amongst these, a tetrabrominated cylindrocyclophane (cylindrocyclophane A_B4_), where the bromination is on the alkylresorcinol motif, has been shown to be 40 times more potent against *M. tuberculosis* than its tetrachlorinated analogue (cylindrocyclophane A_4_).^33^ Interested by the unique effect of this modification, we aimed to investigate the effect a similar transformation upon the resorcinol core of **7 b**. We were able to effect a selective late‐stage bromination of **7 b** using pyridinium tribromide, which yielded tetrabrominated cylindrocyclophane **10** to complete the synthesis for this study.

We screened compounds **1 a**–**c**, **6 a**–**c**, **7 a**–**c** and **8**–**10** for activity against a range of clinical pathogens using an adapted broth microdilution method.^35^ Compounds were tested by using a twofold dilution series in biological duplicate and technical triplicate against *S. aureus* (Newman), epidemic MRSA type 15 (EMRSA‐15), *Serratia marcescens* (Sma12), *Escherichia coli* (Beecham's) and *Pseudomonas aeruginosa* (PA01).

The cyclindrocyclophanes in this work inhibited the growth of *S. aureus* and MRSA (Table 1) selectively, which corroborates the antibacterial activity of cylindrocyclophane natural products reported elsewhere.^18^ Gram‐negative bacteria *S. marcescens*, *E. coli* and *P. aeruginosa* were not susceptible to any of the compounds in this work (minimum inhibitory concentration (MIC) >200 μM). In addition, acetate‐protected monomers **6 a**–**c** and their metathesis products **12 a**–**c** were inactive in all assays, corroborating a previous observation that the resorcinol core is required for biological activity of the cylindrocyclophanes.^30^

**Figure 2:**
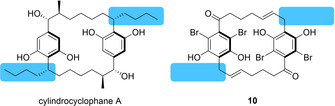
Comparison of cylindrocyclophane A and **10**, highlighting the alkylresorcinol motif in cylindrocyclophane A and the lack thereof in **10**.

The [6.6]cylindrocyclophanes **1 a** and **7 a** exhibited little activity when tested, whereas the [7.7]‐ (**1 b**, **7 b**, **8**, **9** and **10**) and [8.8]cylindrocyclophane (**1 c** and **7 c**) series were more effective in this regard. Doubly oxidized compound **7 b** was the only member of the [7.7]cylindrocyclophanes unable to arrest growth of *S. aureus*. Both [8.8]cylindrocyclophanes **1 c** and **7 c** were effective inhibitors, suggesting that expansion, but not contraction, of the 22‐membered ring may be tolerated by members of this family. An authentic sample of a natural cylindrocyclophane was not available to us at this time, but not one of the analogues in this work exhibited MICs as potent as those reported for cylindrocyclophane A (0.45 μM).^18^ This suggests that the alkylresorcinol motif absent in these analogues imparts activity upon the [7.7]cylindrocyclophane core, although this remains unconfirmed in the absence of a direct comparison (Figure 2).

As such, we focused our study on the natural [7.7] architecture, and in particular the remarkable effect of tetrabrominated compound **10** (MIC 12.5 μM) relative to its unsubstituted congener **7 b** (MIC >200 μM). This result suggests that substitution beyond naturally occurring paracyclophanes might not only be tolerated, but also perhaps a fruitful endeavour in the search for new inhibitors of *S. aureus*. More generally, it may be that bromination, although rarely explored as part of systematic SAR, can improve the activity of related inhibitors of *S. aureus* or other pathogens. We evaluated tetrabrominated macrocycle **10** further against *S. aureus* and determined its minimum bactericidal concentration (MBC) as 25 μM, suggesting a bactericidal mechanism of action for **10**. Cell viability was unaffected by **10** below its MBC but some bacteriostatism was observed at concentrations as low as 6.25 μM.

Many respiratory inhibitors are uncouplers, which dissipate the transmembrane proton gradient to uncouple electron transport from adenosine triphosphate (ATP) synthesis. Uncouplers are typically large, amphiphilic weak acids that can permeate into the cell, ionise once inside, and traverse back out to the cell exterior where it reprotonates and this process repeats. This “short‐circuits” ATP synthesis as a means for proton translocation, dissipating the proton motive force (PMF) and rendering the cell unable to generate energy in the form of ATP. At first glance the structure of **10** lends itself to uncoupling. We sought to characterise its uncoupling ability by measuring oxygen consumption in *S. aureus* using a Clark‐type oxygen microsensor (oxygraph). Treatment of *S. aureus* with **10** (25 μM) was followed by an immediate decrease in oxygen consumption, which suggests that **10** is not an uncoupler – rather that it inhibits some part of the respiratory chain.

We measured susceptibility data for **10** across the pH range 5.0–9.0. Sensitivity to **10** decreased with increasing pH; the MIC increased from 6.25 μM at pH 5.0 to >100 μM at pH 9.0, which supports an mechanism of action involving the transmembrane proton gradient, ΔpH. To corroborate these findings we looked at the ability of sublethal concentrations of **10** to modulate the activity of clinical antibiotics kanamycin and tetracycline. Kanamycin and tetracycline uptake are driven by the electrical potential (Δ*ψ*) and ΔpH, respectively. As such, dissipation of ΔpH increases sensitivity to kanamycin and decreases sensitivity to tetracycline. In line with this, co‐administration of *S. aureus* with **10** (6.25 μM) and the corresponding antibiotic resulted in a modest changes to kanamycin (sensitivity increased ca. twofold) and tetracycline (sensitivity decreased ca. four‐ to eightfold) relative to their untreated controls. These observations suggest that dissipation of ΔpH contributes to the antibacterial activity of **10**, although more work is needed to build on the weak cooperativity seen in these experiments before firm conclusions are drawn.

## Discussion

Both tetrahalogenated cylindrocyclophane analogues (A_4_ and A_B4_) exhibit similar cytotoxicity;^33^ this is an intriguing prospect, as it suggests that cytotoxicity of [7.7]paracyclophanes might not be related to increasing lipophilicity alone, but is still primarily based in the core resorcinol structure.

Based on our findings, we hypothesised that **10** disrupts the *S. aureus* cytoplasmic membrane or cell wall to compromise structure or function. These two targets are more easily accessible than intracellular targets and play crucial roles in cell structure and function (including cellular processes such as resistance, substrate transport, respiration, quorum sensing and energetics), and are conserved across bacteria.^36^ Although the cell wall is an established target inhibited by antibiotics such as β‐lactams and glycopeptides, the cell membrane is relatively unexplored due to concerns around mammalian toxicity.^37^ In line with previous studies, which suggest brominating rigidifies the ore scaffold of membrane‐active macrocycles and increases potency against MRSA,^38^ we thought the chemoselective bromination of cylindrocyclophane **7 b** to afford its halogenated congener **10** would also increase its rigidity and so support a similar mode of action involving membrane disruption. The chemoselective late‐stage bromination using pyridinium tribromide in this study and the scaffolds explored herein may find use in future studies of the cylindrocyclophanes and supramolecular chemistry.^39^, ^40^

## Conclusions

We have reported the development of a divergent synthetic strategy for the study of novel cylindrocyclophane scaffolds. Application of this method enabled us to generate a range of novel macrocycles varying in ring size, oxidation level and functionalisation around the cyclophane core. Antibacterial evaluation of these compounds demonstrated that modification of the cylindrocyclophane natural products can be achieved without total loss of activity, and from these we identified several novel inhibitors of *S. aureus* and methicillin‐resistant *S. aureus*. We have described preliminary structure–activity requirements of these scaffolds, including the requirement for unprotected resorcinols and superiority of the natural [7.7]paracyclophane motif and larger ring sizes. In general, structural simplification of the cylindrocyclophanes was associated with decreased antibacterial activity. Nonetheless, in line with other studies,^14^ we found that bromination increased activity at least eightfold relative to its non‐halogenated congener. This compound (**10**) was less active than has been reported for the cylindrocyclophane natural products,^18^ however bromination of the natural products may yet identify more potent inhibitors than those already known. Detailed profiling of **10** and this family is underway, and developments will be reported in due course.

## Conflict of interest

The authors declare no conflict of interest.

## Supporting information

As a service to our authors and readers, this journal provides supporting information supplied by the authors. Such materials are peer reviewed and may be re‐organized for online delivery, but are not copy‐edited or typeset. Technical support issues arising from supporting information (other than missing files) should be addressed to the authors.

Supplementary
