# Supplementary material for: Divergent Synthesis of Novel Cylindrocyclophanes that Inhibit Methicillin‐Resistant Staphylococcus aureus (MRSA)
Source: ChemMedChem. 2020 Jun 12;15(14):1289–93. doi: 10.1002/cmdc.202000179 (PMC7522682; doi:10.1002/cmdc.202000179)
Supplement: Supplementary file 1 — Supplementary [file CMDC-15-1289-s001.pdf]

ChemMedChem

Supporting Information

# Divergent Synthesis of Novel Cylindrocyclophanes that Inhibit Methicillin-Resistant *Staphylococcus aureus* (MRSA)

Julien J. Freudenreich, Sean Bartlett, Naomi S. Robertson, Sarah L. Kidd, Suzie Forrest, Hannah F. Sore, Warren R. J. D. Galloway, Martin Welch, and David R. Spring*

## Table of Contents

Table of Contents ........ 1

Acronyms and Abbreviations ........ 2

Materials........ 3

Synthesis and Characterisation ........ 5

Biochemical Materials ........ 33

Biochemical Methods........ 34

References........ 36

## Acronyms and Abbreviations

The following trivial names and abbreviations are used in this document.

Grubbs' 2nd generation catalyst, (1,3-bis(2,4,6-trimethylphenyl)-2-imidazolidinylidene)dichloro(phenylmethylene)(tricyclohexylphosphine)ruthenium;

cfu, colony forming units;

$OD_{600}$, optical density (600 nm);

MRSA, methicillin-resistant *Staphylococcus aureus*;

PBS, phosphate-buffered saline;

MHB, Müller-Hinton broth;

LBA, Luria broth agar;

$\lambda$, wavelength;

min, minute(s);

m.p., melting point range;

h, hour(s);

HRMS, high-resolution mass spectrometry;

NMR, nuclear magnetic resonance ;

IR, infrared;

## Materials

Non-aqueous reactions were performed under a constant stream of dry nitrogen using oven-dried glassware. Standard practices were employed when handling moisture- and air-sensitive materials.[1]

Reactions were conducted at ambient temperature unless stated otherwise. All temperatures below 0 °C are that of the external bath. Temperatures of 0 °C were maintained using an ice-water bath; temperatures below 0 °C were maintained using an acetone-ice bath.

$CH_2Cl_2$, ethyl acetate and methanol were distilled from $CaH_2$. Tetrahydrofuran was dried over Na wire and distilled from a mixture of $LiAlH_4$ and $CaH_2$ with triphenylmethane as the indicator. Petroleum ether was distilled before use and refers to the fraction between 40–60 °C. All other reagents and solvents were purchased from commercial vendors and used as received unless stated otherwise

Where possible reactions were monitored by thin layer chromatography (TLC) or low resolution mass spectrometry (LRMS). TLC was performed on glass plates pre-coated with Merck silica gel 60 $F_{254}$; visualisation was by the quenching of ultraviolet (UV) fluorescence ($\lambda_{max}$=254 nm) or by staining with potassium permanganate solution (potassium permanganate, 4.0 g; sodium bicarbonate, 8.0 g; water, 200 mL). Low resolution mass spectra were recorded using liquid chromatography and mass spectrometry (LCMS). LCMS System: Waters ACQUITY H-Class UPLC with an ESCi Multi-Mode Ionisation Waters SQ Detector 2 spectrometer using MassLynx 4.1 software; LC system: solvent A: 2mM $NH_4OAc$ in water/acetonitrile (95:5); solvent B: acetonitrile; solvent C: 2 % formic acid; column: ACQUITY UPLC CSH C18 (2.1 mm ×50 mm, 1.7 µm, 130 Å) at 40 °C; detector: PDA eλ Detector 220–800 nm, interval 1.2 nm.

Yields refer to chromatographically and spectroscopically pure compounds unless stated otherwise. Yields around 100 % are recorded as quantitative. Compounds were purified by chromatography on silica using a Teledyne ISCO Combiflash Rf 200 or preparative high performance liquid chromatography (HPLC). Preparative HPLC system: Agilent 1260 Infinity HPLC with a Supelcosil ABZ+PLUS column (250 mm × 21.2 mm, 5 µm); isocratic solvent system (solvent A: 0.1 % (v/v) TFA in water, solvent B: 0.05 % (v/v) TFA in acetonitrile) over 20 min at a flow rate of 20 mL $min^{-1}$, visualised by UV absorbance ($\lambda_{max}$=254 nm). Melting point ranges (m.p.) were recorded using a Büchi Melting Point B-545 melting point apparatus, and are uncorrected.

Proton magnetic resonance spectra were recorded using an internal deuterium lock (at 298 K unless stated otherwise) on Bruker DPX (400 MHz; $^{1}H$–$^{13}C$ DUL probe), Bruker Avance III HD (400 MHz; Smart probe), Bruker Avance III HD (500 MHz; Smart probe) and Bruker Avance III HD (500 MHz; DCH Cryoprobe) spectrometers. Proton assignments are supported by $^{1}H$–$^{1}H$ COSY, $^{1}H$–$^{13}C$ HSQC or $^{1}H$–$^{13}C$ HMBC spectra, or by analogy. Chemical shifts ($\delta_H$) are quoted in ppm to the nearest 0.01 ppm and are referenced to the residual non- deuterated solvent peak. Coupling constants (*J*) are reported as measured values in Hertz, rounded to the nearest 0.1 Hz. Data are reported as: chemical shift, multiplicity (br, broad; app., apparent; obsc., obscured; s, singlet; d, doublet; t, triplet; q, quartet; qn, quintet; sept, septet; m, multiplet; or a combination thereof), number of nuclei, and coupling constant(s).

Carbon magnetic resonance spectra were recorded using an internal deuterium lock (at 298 K unless stated otherwise) on Bruker DPX (101 MHz), Bruker Avance III HD (101 MHz) and Bruker Avance III HD (126 MHz) spectrometers with broadband proton decoupling. Carbon spectra assignments are supported by DEPT editing, $^{1}H$–$^{13}C$ HSQC or $^{1}H$–$^{13}C$ HMBC spectra, or by analogy. Chemical shifts ($\delta_C$) are quoted in ppm to the nearest 0.1 ppm and are referenced to the deuterated solvent peak.

Infrared (IR) spectra were recorded neat on a Perkin-Elmer Spectrum One spectrometer with a Diamant/KRS5 ATR and internal referencing. Selected absorption maxima ($\nu_{max}$) are reported in wavenumbers ($cm^{-1}$).

High resolution mass spectrometry (HRMS) measurements were recorded on a Bruker Bioapex 4.7e FTICR or a Micromass LCT Premier spectrometer using electrospray mass ionisation. Mass values are quoted within the error limits of ± 5 ppm mass units.

## Synthesis and Characterisation

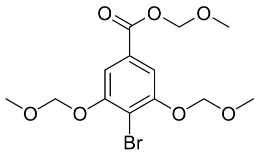

Chloromethyl methyl ether (12.5 mL, 165 mmol) was added dropwise to a solution of 4-bromo-3,5-dihydroxybenzoic acid (11.77 g, 50.0 mmol) in *N*,*N*-diisopropylethylamine:$CH_2CL_2$ (1:1, 100 mL) at 0 °C. The resultant solution was stirred at room temperature for a further 16 h, quenched with saturated aqueous $NH_4Cl$ and twice extracted with $CH_2Cl_2$. The combined organic extract was washed with brine, dried over $MgSO_4$ and evaporated. Purification by chromatography (petroleum ether:ethyl acetate, 1:0→1:1 over 15 min) afforded **13** as a white solid (18.02 g, 49.3 mmol, quantitative).

**m.p.:** 55 – 57 °C.

**$^1$H NMR (500 MHz, $CDCl_3$):** δ 7.52 (s, 2H), 5.47 (s, 2H), 5.31 (s, 4H), 3.54 (s, 3H), 3.53 (s, 6H).

**$^{13}$C NMR (126 MHz, $CDCl_3$):** δ 165.4, 155.0, 130.2, 110.3, 110.1, 95.3, 91.4, 58.1, 56.7.

**IR (cm$^{-1}$):** 2920, 2832, 1724, 1714, 1692, 1429, 1385, 1320, 1301, 1237, 1207, 1150, 1105, 1038, 943, 918, 903, 890, 870, 761.

**HRMS (m/z):** $[M+H]^+$ calculated for $C_{13}H_{18}O_7Br^+$, 365.0236; found, 365.0228.

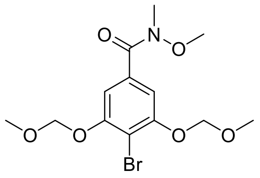

**13** (8.65 g, 23.7 mmol) and Me(MeO)NH·HCl (3.78 g, 38.8 mmol) were dissolved in tetrahydrofuran (100 mL) at -10 °C. A solution of isopropylmagnesium chloride (37.5 mL, 2.0 M in tetrahydrofuran) was added over 20 min to this mixture, ensuring that the temperature did not exceed -5 °C. The reaction mixture was stirred for a further 30 min at -10 °C, quenched with saturated aqueous $NH_4Cl$ and twice extracted with ethyl acetate. The combined organic extract was washed with brine, dried over $MgSO_4$ and evaporated. Purification by chromatography (petroleum ether:ethyl acetate, 1:0→1:4 over 15 min) afforded **3** as a white solid (6.47 g, 17.8 mmol, 75 %).

**m.p.:** 65 – 67 °C.

**$^1$H NMR (400 MHz, $CDCl_3$):** δ 7.17 (s, 2H), 5.27 (s, 4H), 3.60 (s, 3H), 3.52 (s, 6H), 3.33 (s, 3H).

**$^{13}$C NMR (101 MHz, $CDCl_3$):** δ 169.0, 154.7, 134.3, 109.4, 106.5, 95.4, 61.3, 56.6, 33.9.

**IR ($cm^{-1}$):** 2959, 2911, 1646, 1584, 1427, 1383, 1244, 1149, 1104, 1033, 1005, 918, 898, 862, 813.

**HRMS (m/z):** $[M+H]^+$ calculated for $C_{13}H_{19}NO_6Br^+$, 364.0396; found, 364.0379.

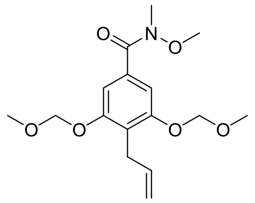

**3** (6.80 g, 18.7 mmol), palladium(II) acetate (126 mg, 0.56 mmol, 3 mol %), 1,1′-bis(di-*tert*-butylphosphino)ferrocene (D*t*BPF, 319 mg, 0.67 mmol, 3.6 mol %) and $K_2CO_3$ (7.75 g, 56.1 mmol) were suspended in tetrahydrofuran (200 mL). Allylboronic acid pinacol ester (8.10 mL, 46.8 mmol) was added to the suspension, and the resultant mixture was stirred at reflux overnight. The reaction was quenched with saturated aqueous $NH_4Cl$ and twice extracted with ethyl acetate. The combined organic extract was washed with brine, dried over $MgSO_4$ and evaporated. Purification by chromatography (petroleum ether:ethyl acetate, 1:0→1:4 over 15 min) afforded **4** as a colourless oil (4.75 g, 14.6 mmol, 78 %).

**$^1$H NMR (500 MHz, CDCl$_3$):** δ 7.10 (s, 2H), 5.94 (ddt, *J* = 17.0 Hz, 10.1 Hz, 6.1 Hz, 1H), 5.19 (s, 4H), 4.98 (ddt, *J* = 17.0 Hz, 3.5 Hz, 1.6 Hz, 1H), 4.95 (ddt, *J* = 10.1 Hz, 3.5 Hz, 1.5 Hz, 1H), 3.62 (s, 3H), 3.47 (obsc. app. dt, *J* = 6.1 Hz, 1.5 Hz, 2H), 3.46 (s, 6H), 3.33 (s, 3H).

**$^{13}$C NMR (126 MHz, CDCl$_3$):** δ 169.9, 155.4, 136.2, 133.3, 121.2, 114.7, 108.1, 94.7, 61.2, 56.2, 34.2, 27.9.

**IR (cm$^{-1}$):** 2935, 2830, 1637, 1578, 1427, 1392, 1374, 1207, 1152, 1120, 1082, 1037, 994, 935, 920, 832.

**HRMS (m/z):** [M+Na]$^+$ calculated for $C_{16}H_{23}NO_6Na^+$, 348.1423; found, 348.1407.

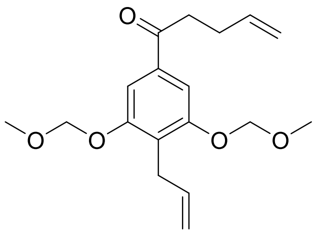

One quarter of a solution of 4-bromo-1-butene (0.65 mL, 6.2 mmol) in tetrahydrofuran (10 mL) was added to a mixture of magnesium turnings (0.45 g, 18.4 mmol) and 1,2-dibromoethane (0.05 mL) in tetrahydrofuran (5 mL) without stirring at room temperature. The reaction was slowly warmed to 40 °C. After 15 min, stirring was begun and the remaining solution of 4-bromo-1-butene was added dropwise. The resultant mixture was stirred for a further 2 h at 40 °C before use.

The Grignard reagent (formed previously) was added dropwise to an ice-cold solution of the Weinreb amide **4** (1.00 g, 3.1 mmol) in tetrahydrofuran (15 mL). The mixture was allowed to warm to room temperature and stirred for a further 2 h. The reaction was quenched with aqueous hydrochloric acid (1 M) and twice extracted with $CH_2Cl_2$. The combined organic extract was washed with brine, dried over $MgSO_4$ and evaporated. Purification by chromatography (petroleum ether:ethyl acetate, 1:0→1:1 over 15 min) afforded **5a** as a white solid (748 mg, 2.33 mmol, 75 %).

**m.p.:** 47 – 49 °C.

**$^1$H NMR (400 MHz, $CDCl_3$):** δ 7.37 (s, 2H), 5.93 (obsc. ddt, *J* = 17.1, 10.2, 6.3 Hz, 1H), 5.90 (obsc. ddt, *J* = 17.1, 10.2, 6.7 Hz, 1H), 5.24 (s, 4H), 5.09 (app. dd, *J* = 17.1, 1.5 Hz, 1H), 5.01 (app. dd, *J* = 10.0, 1.4 Hz, 1H), 4.99 (app. dd, *J* = 16.7, 1.7 Hz, 1H), 4.96 (app. dd, *J* = 9.7, 1.5 Hz, 1H), 3.49 (obsc. app. d, *J* = 6.7 Hz, 2H), 3.48 (s, 6H), 3.03 (t, *J* = 7.4 Hz, 2H), 2.48 (app. q, *J* = 7.0 Hz, 2H).

**$^{13}$C NMR (126 MHz, $CDCl_3$):** δ 198.8, 155.9, 137.6, 136.5, 135.9, 124.1, 115.5, 115.0, 107.7, 94.7, 56.4, 37.9, 28.4, 28.1.

**IR (cm$^{-1}$):** 3078, 2928, 2823, 1676, 1637, 1582, 1443, 1428, 1411, 1394, 1355, 1337, 1313, 1293, 1215, 1157, 1149, 1122, 1081, 1043, 990, 935, 916, 903, 870.

**HRMS (m/z):** $[M+Na]^+$ calculated for $C_{18}H_{24}O_5Na^+$, 343.1516; found, 343.1517.

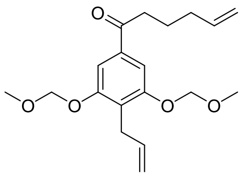

One quarter of a solution of 5-bromo-1-pentene (1.7 mL, 13.5 mmol) in tetrahydrofuran (20 mL) was added to a mixture of magnesium turnings (0.99 g, 40.6 mmol) and 1,2-dibromoethane (0.05 mL) in tetrahydrofuran (10 mL) without stirring at room temperature. The reaction was slowly warmed to 40 °C. After 15 min, stirring was begun and the remaining solution of 5-bromo-1-pentene was added dropwise. The resultant mixture was stirred for a further 2 h at 40 °C before use.

The Grignard reagent (formed above) was added dropwise to an ice-cold solution of the Weinreb amide **4** (2.20 g, 6.76 mmol) in tetrahydrofuran (30 mL). The mixture was allowed to warm to room temperature and stirred for a further 2 h. The reaction was quenched with aqueous hydrochloric acid (1 M) and twice extracted with $CH_2Cl_2$. The combined organic extract was washed with brine, dried over $MgSO_4$ and evaporated. Purification by chromatography (petroleum ether:ethyl acetate, 1:0→1:1 over 15 min) afforded **5b** as a white solid (2.01 g, 6.01 mmol, 89 %).

**m.p.:** 31 – 33 °C.

**$^1$H NMR (500 MHz, $CDCl_3$):** δ 7.36 (s, 2H), 5.93 (ddt, *J* = 16.9, 10.1, 6.2 Hz, 1H), 5.83 (ddt, *J* = 16.9, 10.2, 6.7 Hz, 1H), 5.24 (s, 4H), 5.05 (ddt, *J* = 16.9, 3.5, 1.6 Hz, 1H), 4.99 (ddt, *J* = 10.2, 3.5, 1.6 Hz, 1H), 4.99 (ddt, *J* = 16.9, 3.3, 1.5 Hz, 1H), 4.96 (ddt, *J* = 10.1, 3.3, 1.5 Hz, 1H), 3.49 (obsc. app. dt, *J* = 6.2 Hz, 1.5 Hz, 2H), 3.48 (s, 6H), 2.93 (t, *J* = 7.3 Hz, 2H), 2.18-2.12 (m, 2H), 1,83 (app. qn, *J* = 7.3 Hz, 2H).

**$^{13}$C NMR (126 MHz, $CDCl_3$):** δ 199.7, 155.9, 138.3, 136.6, 135.9, 124.0, 115.3, 115.0, 107.7, 94.7, 56.4, 37.8, 33.3, 28.1, 23.7.

**IR ($cm^{-1}$):** 3079, 2952, 2896, 2820, 1676, 1637, 1581, 1426, 1410, 1393, 1332, 1310, 1291, 1152, 1123, 1109, 1081, 1041, 993, 917, 902, 873, 831.

**HRMS (m/z):** $[M+H]^+$ calculated for $C_{19}H_{27}O_5^+$, 335.1858; found, 335.1842.

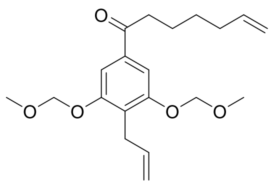

One quarter of a solution of 6-bromo-1-hexene (0.87 mL, 6.2 mmol) in tetrahydrofuran (10 mL) was added to a mixture of magnesium turnings (0.45 g, 18.4 mmol) and 1,2-dibromoethane (0.05 mL) in tetrahydrofuran (5 mL) without stirring at room temperature. The reaction was slowly warmed to 40 °C. After 15 min, stirring was begun and the remaining solution of 6-bromo-1-hexene was added dropwise. The resultant mixture was stirred for a further 2 h at 40 °C before use.

The Grignard reagent (formed above) was added dropwise to an ice-cold solution of the Weinreb amide **4** (1.00 g, 3.1 mmol) in tetrahydrofuran (15 mL). The mixture was allowed to warm to room temperature and stirred for a further 2 h. The reaction was quenched with aqueous hydrochloric acid (1 M) and twice extracted with $CH_2Cl_2$. The combined organic extract was washed with brine, dried over $MgSO_4$ and evaporated. Purification by chromatography (petroleum ether:ethyl acetate, 1:0→1:1 over 15 min) afforded **5c** as a colourless oil (780 mg, 2.24 mmol, 72 %).

**$^1$H NMR (500 MHz, $CDCl_3$):** δ 7.37 (s, 2H), 5.93 (ddt, *J* = 16.8, 10.1, 6.2 Hz, 1H), 5.82 (ddt, *J* = 16.9, 10.2, 6.7 Hz, 1H), 5.24 (s, 4H), 5.02 (obsc. ddt, *J* = 16.9, 3.6, 1.6 Hz, 1H), 4.99 (obsc. ddt, *J* = 16.8, 3.6, 1.8 Hz, 1H), 4.98-4.94 (m, 2H), 3.49 (obsc. app. dt, *J* = 6.2 Hz, 1.6 Hz, 2H), 3.48 (s, 6H), 2.92 (t, *J* = 7.3 Hz, 2H), 2.13–2.08 (m, 2H), 1.74 (app. qn, *J* = 7.4 Hz, 2H), 1.51–1.45 (m, 2H).

**$^{13}$C NMR (126 MHz, $CDCl_3$):** δ 199.7, 155.9, 138.7, 136.6, 135.9, 124.0, 115.0, 114.7, 107.7, 94.7, 56.4, 38.5, 33.7, 28.7, 28.1, 24.1.

**IR (cm$^{-1}$):** 3078, 2928, 2827, 1682, 1638, 1580, 1428, 1392, 1312, 1291, 1208, 1153, 1120, 1084, 1038, 993, 935, 919, 912.

**HRMS (m/z):** $[M+H]^+$ calculated for $C_{20}H_{29}O_5^+$, 349.2015; found, 349.2002.

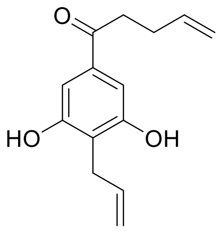


Aqueous hydrochloric acid (1 M, 12 mL) was added to a solution of **5a** (692 mg, 2.16 mmol) in methanol (24 mL). The homogenous mixture was stirred at 60 °C for 1–3 h until complete deprotection. The reaction was concentred under reduced pressure and extracted three times with ethyl acetate. The combined organic extract was washed with brine, dried over $MgSO_4$ and evaporated. Purification by chromatography (petroleum ether:ethyl acetate, 1:0→1:4 over 15 min) afforded **14a** as a white solid (499 mg, 2.15 mmol, quantitative).

**m.p.:** 109 – 111 °C.

**$^1$H NMR (400 MHz, $CDCl_3$):** δ 7.14 (s, 2H), 6.00 (ddt, *J* = 17.0, 10.2, 6.1 Hz, 1H), 5.82 (s, 2H), 5.87 (obsc. ddt, *J* = 17.0, 10.3, 6.7 Hz, 1H), 5.18 (obsc. ddd, *J* = 17.2, 3.3, 1.6 Hz, 1H), 5.17 (obsc. ddd, *J* = 10.1, 3.0, 1.5 Hz, 1H), 5.08 (ddd, *J* = 17.1, 3.2, 1.6 Hz, 1H), 5.01 (ddd, *J* = 10.3, 2.8, 1.3 Hz, 1H), 3.53 (dt, *J* = 6.1, 1.4 Hz, 2H), 3.01 (t, *J* = 7.4 Hz, 2H), 2.51–2.43 (m, 2H).

**$^{13}$C NMR (101 MHz, $CDCl_3$):** δ 200.0, 155.5, 137.2, 136.4, 135.2, 118.0, 116.7, 115.6, 108.2, 37.8, 28.3, 28.0.

**IR ($cm^{-1}$):** 3512, 3456, 3345, 3284, 3082, 3001, 2980, 2926, 1672, 1652, 1591, 1425, 1380, 1366, 1331, 1322, 1291, 1193, 1182, 1154, 1112, 1046, 1023, 1000, 993, 910, 860.

**HRMS (m/z):** $[M\text{-}H]^-$ calculated for $C_{14}H_{15}O_3^-$, 231.1021; found, 231.1033.

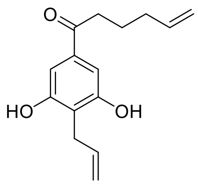

Aqueous hydrochloric acid (1 M, 50 mL) was added to a solution of **5b** (2.78 g, 8.31 mmol) in methanol (100 mL). The homogenous mixture was stirred at 60 °C for 1–3 h until complete deprotection. The reaction was concentred under reduced pressure and extracted three times with ethyl acetate. The combined organic extract was washed with brine, dried over $MgSO_4$ and evaporated. Purification by chromatography (petroleum ether:ethyl acetate, 1:0→1:4 over 15 min) afforded **14b** as a white solid (2.04 g, 8.28 mmol, quantitative).

**m.p.:** 82 – 84 °C.

**$^1$H NMR (400 MHz, $CDCl_3$):** δ 7.16 (s, 2H), 6.00 (ddt, *J* = 16.3, 10.2, 6.1 Hz, 1H), 5.82 (s, 2H), 5.80 (obsc. ddt, *J* = 16.9, 10.2, 6.7 Hz, 1H), 5.22–5.15 (m, 2H), 5.06–4.97 (m, 2H), 3.54 (app. d, *J* = 6.1 Hz, 2H), 2.91 (t, *J* = 7.4 Hz, 2H), 2.14 (app. q, *J* = 7.1 Hz, 2H), 1.82 (app. qn, *J* = 7.4 Hz, 2H).

**$^{13}$C NMR (101 MHz, $CDCl_3$):** δ 201.0, 155.5, 138.1, 136.5, 135.2, 118.0, 116.7, 115.6, 108.2, 37.8, 33.3, 28.0, 23.5.

**IR ($cm^{-1}$):** 3534, 3410, 3345, 2922, 1668, 1652, 1591, 1423, 1408, 1368, 1340, 1322, 1312, 1193, 1154, 1047, 998, 989, 913, 849, 828.

**HRMS (m/z):** $[M-H]^-$ calculated for $C_{15}H_{17}O_3^-$, 245.1178; found, 245.1186.

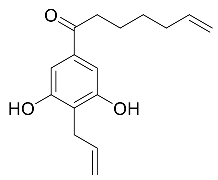

Aqueous hydrochloric acid (1 M, 15 mL) was added to a solution of **5c** (780 mg, 2.24 mmol) in methanol (30 mL). The homogenous mixture was stirred at 60 °C for 1–3 h until complete deprotection. The reaction was concentred under reduced pressure and extracted three times with ethyl acetate. The combined organic extract was washed with brine, dried over $MgSO_4$ and evaporated. Purification by chromatography (petroleum ether:ethyl acetate, 1:0→1:4 over 15 min) afforded **14c** as a white solid (580 mg, 2.23 mmol, quantitative).

**m.p.:** 80 – 82 °C.

**$^1$H NMR (500 MHz, $CDCl_3$):** δ 7.14 (s, 2H), 6.00 (ddt, *J* = 17.0, 10.1, 6.1 Hz, 1H), 5.81 (ddt, *J* = 16.9, 10.2, 6.7 Hz, 1H), 5.72 (s, 2H), 5.19 (app. ddd, *J* = 17.2, 3.4, 1.7 Hz, 1H), 5.17 (app. dq, *J* = 10.0, 1.5 Hz, 1H), 5.01 (app. ddd, *J* = 17.1, 3.6, 1.6 Hz, 1H), 4.95 (ddt, *J* = 10.2, 2.2, 1.2 Hz, 1H), 3.53 (app. dt, *J* = 6.1, 1.6 Hz, 2H), 2.90 (t, *J* = 7.4 Hz, 2H), 2.12–2.07 (m, 2H), 1.73 (app. q, *J* = 7.5 Hz, 2H), 1.49-1.43 (m, 2H).

**$^{13}$C NMR (126 MHz, $CDCl_3$):** δ 200.9, 155.5, 138.6, 136.5, 135.2, 117.9, 116.8, 114.8, 108.2, 38.5, 33.7, 28.7, 28.0, 24.0.

**IR (cm$^{-1}$):** 3393, 3078, 2928, 2858, 1673, 1659, 1639, 1587, 1423, 1410, 1373, 1359, 1342, 1297, 1180, 1154, 1107, 1023, 990, 906, 868, 848.

**HRMS (m/z):** $[M-H]^-$ calculated for $C_{16}H_{19}O_3^-$, 259.1334; found, 259.1346.

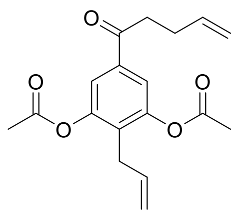

Acetyl chloride (0.642 mL, 8.85 mmol) was added dropwise to an ice-cold solution of **14a** (466 mg, 2.01 mmol) and triethylamine (1.245 mL, 8.84 mmol) in $CH_2Cl_2$ (40 mL). The resultant mixture was stirred at room temperature overnight. The reaction was quenched with saturated aqueous $NH_4Cl$ and twice extracted with ethyl acetate. The combined organic extract was washed with brine, dried over $MgSO_4$ and evaporated. Purification by chromatography (petroleum ether:ethyl acetate, 1:0→1:1 over 15 min) afforded **6a** as a white solid (417 mg, 1.32 mmol, 66 %).

**m.p.:** 40 – 42 °C.

**$^1$H NMR (500 MHz, $CDCl_3$):** δ 7.57 (s, 2H), 5.87 (ddt, *J* = 16.8, 10.2, 6.5 Hz, 1H), 5.75 (ddt, *J* = 16.4, 10.1, 6.2 Hz, 1H), 5.08 (ddt, *J* = 17.1, 3.0, 1.5 Hz, 1H), 5.03 (obsc. ddt, *J* = 10.3, 3.0, 1.5 Hz, 1H), 5.01 (obsc. ddt, *J* = 10.9, 2.8, 1.5 Hz, 1H), 5.00 (obsc. ddt, *J* = 17.1, 3.0, 1.6 Hz, 1H), 3.28 (app. dt, *J* = 6.2, 1.5 Hz, 2H), 3.01 (t, *J* = 7.3 Hz, 2H), 2.50–2.44 (m, 2H), 2.32 (s, 6H).

**$^{13}$C NMR (126 MHz, $CDCl_3$):** δ 197.2, 169.0, 150.1, 137.2, 136.3, 134.0, 130.4, 120.1, 116.5, 115.6, 37.9, 29.6, 28.0, 21.0.

**IR (cm$^{-1}$):** 3088, 3010, 2917, 1759, 1689, 1639, 1574, 1414, 1367, 1314, 1301, 1295, 1206, 1179, 1143, 1111, 1031, 991, 916, 890.

**HRMS (m/z):** $[M+Na]^+$ calculated for $C_{18}H_{20}O_5Na^+$, 339.1208; found, 339.1194.

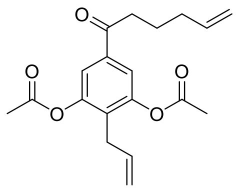

Acetyl chloride (2.24 mL, 30.9 mmol) was added dropwise to an ice-cold solution of **14b** (1.73 g, 7.02 mmol) and triethylamine (4.35 mL, 30.9 mmol) in $CH_2Cl_2$ (150 mL). The resultant mixture was stirred at room temperature overnight. The reaction was quenched with saturated aqueous $NH_4Cl$ and twice extracted with ethyl acetate. The combined organic extract was washed with brine, dried over $MgSO_4$ and evaporated. Purification by chromatography (petroleum ether:ethyl acetate, 1:0→1:1 over 15 min) afforded **6b** as a white solid (2.03 g, 6.14 mmol, 87 %).

**m.p.:** 40 – 42 °C.

**$^1$H NMR (400 MHz, CDCl$_3$):** δ 7.56 (s, 2H), 5.80 (obsc. ddt, *J* = 16.9, 10.2, 6.7 Hz, 1H), 5.75 (obsc. ddt, *J* = 16.5, 10.2, 6.2 Hz, 1H), 5.07–4.97 (m, 4H), 3.28 (app. dt, *J* = 6.2 Hz, 1.4 Hz, 2H), 2.91 (t, *J* = 7.3 Hz, 2H), 2.32 (s, 6H), 2.14 (app. q, *J* = 7.1 Hz, 2H), 1.83 (app. qn, *J* = 7.3 Hz, 2H).

**$^{13}$C NMR (101 MHz, CDCl$_3$):** δ 197.9, 169.0, 150.1, 138.1, 136.5, 134.0, 130.3, 120.1, 116.5, 115.5, 37.8, 33.2, 29.6, 23.1, 21.0.

**IR (cm$^{-1}$):** 3074, 2943, 1760, 1688, 1637, 1573, 1433, 1417, 1368, 1287, 1208, 1179, 1147, 1111, 1032, 1011, 989, 904, 891, 871.

**HRMS (m/z):** $[M+Na]^+$ calculated for $C_{19}H_{22}O_5Na^+$, 353.1365; found, 353.1353.

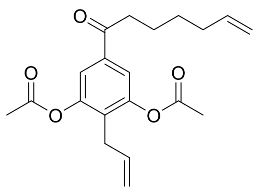

Acetyl chloride (0.281 mL, 3.87 mmol) was added dropwise to an ice-cold solution of **14c** (230 mg, 0.88 mmol) and triethylamine (0.545 mL, 3.87 mmol) in $CH_2Cl_2$ (20 mL). The resultant mixture was stirred at room temperature overnight. The reaction was quenched with saturated aqueous $NH_4Cl$ and twice extracted with ethyl acetate. The combined organic extract was washed with brine, dried over $MgSO_4$ and evaporated. Purification by chromatography (petroleum ether:ethyl acetate, 1:0→1:1 over 15 min) afforded **6c** as a colourless oil (225 mg, 0.65 mmol, 74 %).

**$^{1}$H NMR (500 MHz, CDCl$_3$):** δ 7.56 (s, 2H), 5.81 (obsc. ddt, *J* = 16.9, 10.2, 6.7 Hz, 1H), 5.75 (obsc. ddt, *J* = 17.0, 10.1, 6.2 Hz, 1H), 5.03 (app. obsc qd, *J* = 10.1, 1.5 Hz, 1H), 5.02 (app. obsc. ddd, *J* = 17.1, 3.9, 1.6 Hz, 1H), 5.00 (app. obsc qd, *J* = 16.9, 1.6 Hz, 1H), 4.96 (ddt, *J* = 10.2, 2.2, 1.2 Hz, 1H), 3.28 (app. dt, *J* = 6.2, 1.5 Hz, 2H), 2.91 (t, *J* = 7.3 Hz, 2H), 2.32 (s, 6H), 2.12–2.07 (m, 2H), 1.74 (app. q, *J* = 7.5 Hz, 2H), 1.49-1.43 (m, 2H).

**$^{13}$C NMR (126 MHz, CDCl$_3$):** δ 198.0, 169.0, 150.1, 138.6, 136.5, 134.0, 130.3, 120.1, 116.5, 114.8, 38.5, 33.7, 29.6, 28.6, 23.6, 21.0.

**IR (cm$^{-1}$):** 2928, 2857, 1768, 1686, 1639, 1573, 1415, 1368, 1291, 1204, 1177, 1106, 1031, 993, 911, 890.

**HRMS (m/z):** $[M+H]^+$ calculated for $C_{20}H_{25}O_5^+$, 345.1702; found, 345.1686.

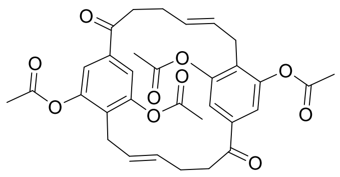

Grubbs' 2nd generation catalyst (49.2 mg, 0.06 mmol, 5 mol %) was added to a solution of **6a** (367 mg, 1.16 mmol) in $CH_2Cl_2$ (230 mL). The reaction mixture heated at reflux for 20 h, cooled and then evaporated. Purification by preparative HPLC (50–90 % B) afforded **15a** (27.2 mg, 0.047 mmol, 4 %) and **12a** (164 mg, 0.19 mmol, 16 %) as white solids.

**$^1$H NMR (500 MHz, $CDCl_3$):** δ 7.35 (s, 4H), 5.39 (app. obsc. t, *J* = 5.1 Hz, 2H), 5.38 (app. obsc. t, *J* = 5.4 Hz, 2H), 3.02 (d, *J* = 4.62 Hz, 4H), 2.83–2.79 (m, 4H), 2.51–2.46 (m, 4H), 2.31 (s, 12H).

**$^{13}$C NMR (126 MHz, $CDCl_3$):** δ 199.7, 169.0, 149.4, 136.8, 130.9, 130.5, 127.5, 120.1, 37.0, 28.0, 27.7, 21.0.

**IR (cm$^{-1}$):** 2964, 2918, 2849, 1766, 1678, 1568, 1415, 1370, 1339, 1292, 1192, 1169, 1142, 1037, 967, 909, 889.

**HRMS (m/z):** [M+Na]$^+$ calculated for $C_{32}H_{32}O_{10}Na^+$, 599.1893; found, 599.1876.

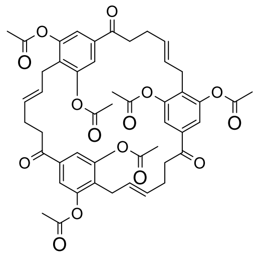

**$^{1}$H NMR (400 MHz, $CDCl_3$):** δ 7.55–7.49 (app. 4s, 6H), 5.54 (dt, *J* = 5.2, 3.8 Hz, 1H), 5.52–5.34 (m, 4H), 5.32 (app. br. t, *J* = 3.6 Hz, 1H), 3.23–3.10 (m, 6H), 3.06–2.82 (m, 6H), 2.45–2.30 (m, 6H), 2.29-2.13 (app. 4s, 18H).

**$^{13}$C NMR (101 MHz, $CDCl_3$):** δ 197.5, 197.5, 197.3, 169.2, 169.1, 169.0, 150.0, 150.0, 150.0, 136.5, 136.3, 136.1, 136.0, 131.1, 130.9, 130.4, 130.3, 130.0, 127.5, 127.4, 126.4, 126.3, 120.2, 120.1, 119.9, 119.9, 38.8, 38.7, 38.2, 38.2, 28.6, 28.3, 28.2, 26.6, 26.2, 26.2, 21.0, 20.9, 20.8, 20.8.

**IR ($cm^{-1}$):** 2924, 2857, 1766, 1686, 1573, 1416, 1368, 1285, 1185, 1142, 1082, 1034, 970, 891, 852, 802.

**HRMS (m/z):** $[M+Na]^+$ calculated for $C_{48}H_{48}O_{15}Na^+$, 887.2885; found, 887.2855.

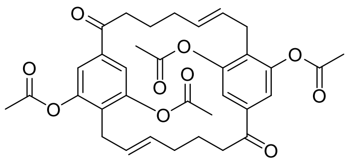

Grubbs' 2nd generation catalyst (98 mg, 0.12 mmol, 5 mol %) was added to a solution of **6b** (759 mg, 2.30 mmol) in $CH_2Cl_2$ (350 mL). The reaction mixture heated at reflux for 20 h, cooled and then evaporated. Precipitation from $CH_2Cl_2$/methanol afforded **15b** as a white solid (426 mg, 0.71 mmol, 61 %).

**$^1$H NMR (400 MHz, $CDCl_3$):** δ 7.36 (s, 4H), 5.41–5.23 (m, 4H), 3.12 (d, *J* = 6.7 Hz, 4H), 2.77 (t, *J* = 7.1 Hz, 4H), 2.30 (s, 12H), 2.15–2.07 (m, 4H), 1.86–1.77 (m, 4H).

**$^{13}$C NMR (101 MHz, $CDCl_3$):** δ 197.6, 168.9, 149.6, 136.3, 132.0, 131.2, 127.7, 119.8, 36.0, 31.0, 29.0, 21.0, 20.9.

**IR (cm$^{-1}$):** 2933, 1769, 1685, 1571, 1415, 1368, 1319, 1275, 1197, 1177, 1147, 1137, 1098, 1072, 1042, 1026, 961, 931, 891.

**HRMS (m/z):** $[M+H]^+$ calculated for $C_{34}H_{37}O_{10}^+$, 605.2387; found, 605.2369.

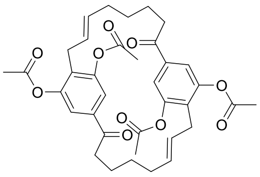

Grubbs' 2nd generation catalyst (23 mg, 0.027 mmol, 5 mol %) was added to a solution of **6c** (184 mg, 0.53 mmol) in $CH_2Cl_2$ (85 mL). The reaction mixture heated at reflux for 20 h, cooled and then evaporated. Purification by preparative HPLC (50–90 % B) afforded **15c** (16.9 mg, 0.027 mmol, 10 %), **12c.1** (10.4 mg, 0.011 mmol, 6 %) and **12c.2** (10.2 mg, 0.011 mmol, 6 %) as white solids.

**$^1$H NMR (500 MHz, CDCl$_3$):** δ 7.51 (s, 4H), 5.43 (app. t, *J* = 3.5 Hz, 2H), 5.32 (app. t, *J* = 3.4 Hz, 2H), 3.15 (d, *J* = 3.9 Hz, 4H), 2.81 (t, *J* = 7.5 Hz, 4H), 2.16 (s, 12H), 2.02 (app. br. dd, *J* = 11.1, 6.6 Hz, 4H), 1.75 (app. obsc. q, *J* = 7.5 Hz, 4H), 1.45 (app. q, *J* = 7.1 Hz, 4H).

**$^{13}$C NMR (126 MHz, CDCl$_3$):** δ 200.0, 168.7, 149.8, 135.8, 130.2, 130.1, 127.6, 120.3, 38.5, 32.0, 28.9, 28.1, 25.7, 20.8.

**IR (cm$^{-1}$):** 2935, 2853, 1765, 1678, 1573, 1416, 1368, 1293, 1173, 1135, 1033, 969, 891, 852.

**HRMS (m/z):** $[M+H]^+$ calculated for $C_{36}H_{41}O_{10}^+$, 633.2700; found, 633.2678.

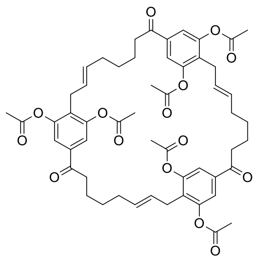

**$^{1}$H NMR (500 MHz, $CDCl_3$):** δ 7.51 (s, 6H), 5.34 (app. obsc. t, *J* = 4.0 Hz, 3H), 5.34 (app. obsc. t, *J* = 4.9 Hz, 3H), 3.18 (d, *J* = 3.6 Hz, 6H), 2.85 (t, *J* = 7.2 Hz, 6H), 2.30 (s, 18H), 1.98 (app. dd, *J* = 11.5, 6.5 Hz, 6H), 1.64 (app. q, *J* = 7.4 Hz, 6H), 1.34 (app. q, *J* = 7.5 Hz, 6H).

**$^{13}$C NMR (126 MHz, $CDCl_3$):** δ 198.0, 169.0, 150.0, 136.2, 132.1, 131.1, 125.6, 120.0, 38.5, 32.2, 28.9, 28.3, 23.2, 21.0.

**IR ($cm^{-1}$):** 2933, 2857, 1766, 1683, 1572, 1415, 1368, 1291, 1172, 1135, 1033, 969, 922, 892.

**HRMS (m/z):** $[M+Na]^+$ calculated for $C_{54}H_{60}O_{15}Na^+$, 971.3830; found, 971.3878.

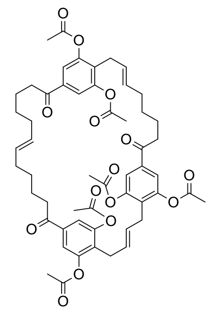

**$^{1}$H NMR (500 MHz, CDCl$_3$):** δ 7.55 (s, 2H), 7.53 (s, 2H), 7.51 (s, 2H), 5.42–5.38 (m, 2H), 5.37–5.32 (m, 4H), 3.20 (d, *J* = 3.8 Hz, 2H), 3.15 (d, *J* = 3.5 Hz, 4H), 2.91 (obsc. t, *J* = 7.2 Hz, 2H), 2.88 (obsc. t, *J* = 7.2 Hz, 2H), 2.84 (t, *J* = 7.3 Hz, 2H), 2.31 (s, 6H), 2.13 (s, 12H), 2.05–1.95 (m, 6H), 1.71 (app. obsc. sept, *J* = 7.2 Hz, 4H), 1.64 (app. obsc. q, *J* = 7.5 Hz, 2H), 1.42 (app. q, *J* = 7.6 Hz, 4H), 1.34 (app. q, *J* = 7.5 Hz, 2H).

**$^{13}$C NMR (126 MHz, CDCl$_3$):** δ 198.2, 198.2, 169.0, 168.9, 168.9, 150.0, 136.4, 136.3, 136.1, 132.1, 131.0, 130.5, 130.4, 130.2, 130.1, 127.6, 127.5, 125.6, 120.1, 119.9, 119.8, 38.6, 38.6, 38.5, 38.4, 32.2, 32.2, 32.1, 29.3, 29.2, 28.9, 28.5, 28.2, 23.4, 23.4, 23.2, 21.0, 20.8.

**IR (cm$^{-1}$):** 2934, 2857, 1765, 1683, 1573, 1415, 1367, 1289, 1173, 1079, 1033, 969, 922, 891, 852.

**HRMS (m/z):** [M+Na]$^+$ calculated for $C_{54}H_{60}O_{15}Na^+$, 971.3830; found, 971.3840.

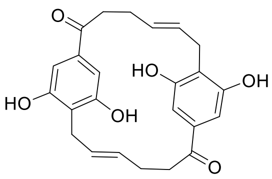


NaOH (12 equivalents) was added to a homogeneous solution of **15a** (20 mg, 0.0347 mmol) in methanol:$CH_2Cl_2$:$H_2O$ (4:1:1, 18 mL). The reaction mixture was stirred at room temperature for 1 h and then the solvent was evaporated. Purification by preparative HPLC (50–90 % B) afforded **7a** (12.4 mg, 0.030 mmol, 87 %) as a white solid.

**$^1$H NMR (500 MHz, $(CD_3)_2SO$):** δ 9.08 (s, 4H), 6.65 (s, 4H), 5.54 (dt, *J* = 15.3, 5.5 Hz, 2H), 5.01 (br dt, *J* = 15.2, 6.9 Hz, 2H), 3.12 (d, *J* = 5.0 Hz, 4H), 2.64–2.60 (m, 4H), 2.36–2.30 (m, 4H).

**$^{13}$C NMR (126 MHz, $(CD_3)_2SO$):** δ 200.9, 155.4, 136.0, 128.4, 127.3, 118.6, 106.3, 35.8, 28.9, 25.2.

**IR (cm$^{-1}$):** 3408, 2924, 2848, 1655, 1593, 1583, 1453, 1420, 1352, 1314, 1289, 1277, 1187, 1132, 1047, 1041, 976, 862, 853, 841, 831.

**HRMS (m/z):** $[M+H]^+$ calculated for $C_{24}H_{25}O_6^+$, 409.1651; found, 409.1639.

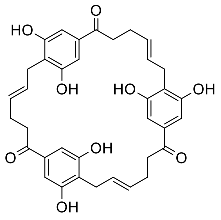

NaOH (12 equivalents) was added to a homogeneous solution of **12a** (20 mg, 0.0231 mmol) in methanol:$CH_2Cl_2$:$H_2O$ (4:1:1, 18 mL). The reaction mixture was stirred at room temperature for 1 h and then the solvent was evaporated. Purification by preparative HPLC (50–90 % B) afforded **16a** (11.9 mg, 0.0194 mmol, 84 %) as a white solid.

**$^1$H NMR (500 MHz, $(CD_3)_2SO$):** δ 9.41 (s, 1H), 9.40 (s, 3H), 9.35 (s, 1H), 9.33 (s, 1H), 6.84 (s, 1H), 6.81 (s, 1H), 6.80 (s, 3H), 6.75 (s, 1H), 5.51–5.35 (m, 6H), 3.22–3.13 (m, 6H), 2.86 (t, *J* = 6.9 Hz, 2H), 2.79 (obsc. t, *J* = 7.1 Hz, 3H), 2.76 (obsc. t, *J* = 7.1 Hz, 1H), 2.27–2.15 (m, 6H).

**$^{13}$C NMR (126 MHz, $(CD_3)_2SO$):** δ 198.5, 198.4, 198.2, 156.0, 135.2, 135.1, 135.1, 129.5, 129.4, 128.9, 128.8, 127.5, 127.4, 126.9, 126.7, 118.9, 118.9, 118.9, 105.8, 105.7, 105.6, 37.9, 37.7, 37.5, 26.3, 26.1, 26.0.

**IR (cm$^{-1}$):** 3335, 2919, 1660, 1582, 1421, 1345, 1324, 1180, 1148, 1086, 1034, 970, 852, 839.

**HRMS (m/z):** $[M+H]^+$ calculated for $C_{36}H_{37}O_9^+$, 613.2438; found, 613.2413.

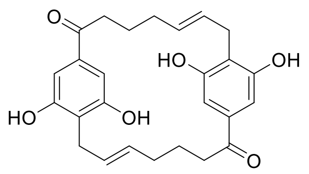


NaOH (12 equivalents) was added to a homogeneous solution of **14b** (20 mg, 0.0331 mmol) in methanol:$CH_2Cl_2$:$H_2O$ (4:1:1, 18 mL). The reaction mixture was stirred at room temperature for 1 h, and then the solvent was evaporated. Purification by preparative HPLC (50–90 % B) afforded **7b** (12.9 mg, 0.0296 mmol, 89 %) as a white solid.

**$^1$H NMR (500 MHz, $(CD_3)_2SO$):** δ 9.22 (s, 4H), 6.73 (s, 4H), 5.29 (app. dt, *J* = 15.1, 7.0 Hz, 2H), 5.13 (app. dt, *J* = 15.1, 6.4 Hz, 2H), 3.18 (d, *J* = 6.4 Hz, 4H), 2.77 (t, *J* = 6.6 Hz, 4H), 2.03–1.96 (m, 4H), 1.66–1.59 (m, 4H).

**$^{13}$C NMR (126 MHz, $(CD_3)_2SO$):** δ 198.8, 155.7, 135.2, 129.8, 128.6, 118.6, 105.4, 34.8, 30.2, 26.3, 21.7.

**IR (cm$^{-1}$):** 3403, 2932, 2849, 1666, 1590, 1423, 1377, 1346, 1330, 1206, 1191, 1172, 1152, 1141, 1096, 1021, 983, 963, 865, 826.

**HRMS (m/z):** $[M+Na]^+$ calculated for $C_{26}H_{28}O_6Na^+$, 459.1778; found, 459.1760.

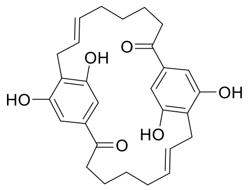


NaOH (12 equivalents) was added to a homogeneous solution of **14c** (20 mg, 0.0316 mmol) in methanol:$CH_2Cl_2$:$H_2O$ (4:1:1, 18 mL). The reaction mixture was stirred at room temperature for 1 h, and then the solvent was evaporated. Purification by preparative HPLC (50–90 % B) afforded **7c** (12.8 mg, 0.0276 mmol, 87 %) as a white solid.

**$^{1}$H NMR (400 MHz, $(CD_3)_2SO$):** δ 9.41 (s, 4H), 6.80 (s, 4H), 5.46 (app. br. s, 2H), 5.23 (app. t, *J* = 3.5 Hz, 2H), 3.21–3.16 (m, 4H), 2.67 (t, *J* = 6.4 Hz, 4H), 1.89-1.80 (m, 4H), 1.56 (app. q, *J* = 6.9 Hz, 4H), 1.16 (app. q, *J* = 7.1 Hz, 4H).

**$^{13}$C NMR (101 MHz, $(CD_3)_2SO$):** δ 200.4, 155.9, 135.1, 129.8, 127.2, 119.6, 106.2, 36.7, 31.9, 28.6, 25.6, 25.5.

**IR (cm$^{-1}$):** 3397, 2924, 2854, 1670, 1583, 1422, 1370, 1345, 1294, 1180, 1139, 1095, 1033, 967, 855.

**HRMS (m/z):** $[M+H]^+$ calculated for $C_{28}H_{33}O_6^+$, 465.2277; found, 465.2262.

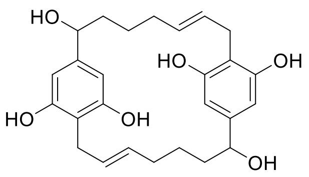

Sodium borohydride (8.3 mg, 0.22 mmol) was added to a solution of **7b** (20 mg, 0.046 mmol) in methanol (5 mL). The reaction was stirred at room temperature for 30 min, then quenched with saturated aqueous $NH_4Cl$ and twice extracted with ethyl acetate. The combined organic extract was washed with brine, dried over $MgSO_4$ and evaporated. Purification by preparative HPLC (30–80 % B) afforded **8** (11.2 mg, 0.0254 mmol, 55 %) as a white solid.

**$^1$H NMR (500 MHz, $(CD_3)_2SO$):** δ 8.76 (s, 4H), 8.76 (s, 4H), 6.14 (s, 8H), 5.36 (app. dt, *J* = 15.2, 6.4 Hz, 4H), 5.13 (app. dt, *J* = 15.2, 6.6 Hz, 4H), 4.85 (d, *J* = 3.7 Hz, 2H), 4.84 (d, *J* = 3.7 Hz, 2H), 4.26 (br td, *J* = 5.9, 3.7 Hz, 2H), 4.20 (br td, *J* = 6.4, 3.7 Hz, 2H), 3.11–3.00 (m, 8H), 1.96–1.68 (m, 8H), 1.55–1.37 (m, 8H), 1.10–1.00 (m, 2H), 1.00–0.92 (m, 2H), 0.92–0.82 (m, 2H), 0.82–0.71 (m, 2H).

**$^{13}$C NMR (126 MHz, $(CD_3)_2SO$):** δ 155.1, 144.0, 143.9, 128.8, 128.8, 128.3, 128.2, 111.6, 111.6, 104.1, 72.6, 72.1, 38.7, 38.5, 32.7, 32.7, 25.7, 25.3, 24.8.

**IR (cm$^{-1}$):** 3314, 2931, 2853, 1625, 1594, 1432, 1339, 1309, 1258, 1194, 1172, 1152, 1030, 1006, 971, 838.

**HRMS (m/z):** $[M+Na]^+$ calculated for $C_{26}H_{32}O_6Na^+$, 463.2091; found, 463.2091.

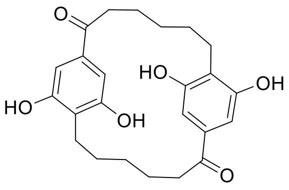


Palladium on barium sulphate (5 % Pd basis, 1.0 mg, 10 wt %) was added to a solution of **7a** (10 mg, 0.0245 mmol) in acetone (5 mL). A $H_2$ atmosphere was applied and the reaction stirred at room temperature overnight. The mixture was filtered through a small pad of Celite and the filtrate evaporated. Purification by preparative HPLC (30–80 % B) afforded **1a** (6.1 mg, 0.0148 mmol, 60 %) as a white solid.

**$^{1}$H NMR (500 MHz, $(CD_3)_2SO$):** δ 9.14 (s, 4H), 6.66 (s, 4H), 2.60–2.55 (m, 4H), 2.52 (obsc. t, *J* = 6.7 Hz, 4H), 1.64–1.57 (m, 4H), 1.41 (app. q, *J* = 6.9 Hz, 4H), 1.14 (app. q, *J* = 7.2 Hz, 4H).

**$^{13}$C NMR (126 MHz, $(CD_3)_2SO$):** δ 199.9, 155.9, 134.8, 120.5, 105.9, 36.3, 27.1, 26.7, 25.3, 22.5.

**IR ($cm^{-1}$):** 3411, 3190, 2930, 2858, 1651, 1587, 1418, 1357, 1340, 1261, 1236, 1202, 1125, 1093, 1019, 980, 927, 869, 836, 826.

**HRMS (m/z):** $[M+H]^+$ calculated for $C_{24}H_{29}O_6^+$, 413.1959; found, 413.1966.

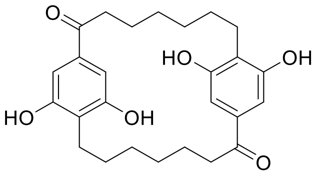


Palladium on barium sulphate (5 % Pd basis, 1.0 mg, 10 wt %) was added to a solution of **7b** (10 mg, 0.0229 mmol) in acetone (5 mL). A $H_2$ atmosphere was applied and the reaction stirred at room temperature overnight. The mixture was filtered through a small pad of Celite and the filtrate evaporated. Purification by preparative HPLC (20–80 % B) afforded **1b** (3.5 mg, 0.0079 mmol, 35 %) as a white solid.

**$^1$H NMR (500 MHz, $(CD_3)_2SO$):** δ 9.20 (s, 4H), 6.70 (s, 4H), 2.58 (obsc. t, *J* = 6.8 Hz, 4H), 2.56 (obsc. t, *J* = 6.4 Hz, 4H), 1.50 (app. obsc. qn, *J* = 6.8 Hz, 4H), 1.54–1.44 (m, 4H), 1.20 (app. qn, *J* = 7.1 Hz, 4H), 1.01 (app. qn, *J* = 7.6 Hz, 4H).

**$^{13}$C NMR (126 MHz, $(CD_3)_2SO$):** δ 199.7, 156.2, 134.8, 120.1, 105.5, 36.9, 27.4, 26.9, 26.2, 22.9, 22.0.

**IR (cm$^{-1}$):** 3370, 2932, 2861, 1673, 1649, 1584, 1421, 1368, 1348, 1329, 1189, 1143, 1084, 1014, 917, 867, 837.

**HRMS (m/z):** $[M+Na]^+$ calculated for $C_{26}H_{32}O_6Na^+$, 463.2091; found, 463.2091.

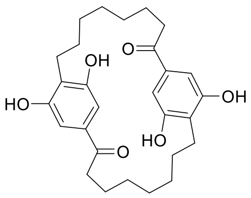


Palladium on barium sulphate (5 % Pd basis, 1.0 mg, 10 wt %) was added to a solution of **7c** (10 mg, 0.0215 mmol) in acetone (5 mL). A $H_2$ atmosphere was applied and the reaction stirred at room temperature overnight. The mixture was filtered through a small pad of Celite and the filtrate evaporated. Purification by preparative HPLC (40–80 % B) afforded **1c** (4.7 mg, 0.010 mmol, 47 %) as a white solid.

**$^{1}$H NMR (400 MHz, $(CD_3)_2SO$):** δ 9.31 (s, 4H), 6.80 (s, 4H), 2.61 (t, *J* = 7.0 Hz, 4H), 2.55 (t, *J* = 6.3 Hz, 4H), 1.43–1.52 (m, 8H), 1.05–1.20 (m, 12H).

**$^{13}$C NMR (101 MHz, $(CD_3)_2SO$):** δ 200.0, 156.3, 134.9, 120.9, 105.9, 37.0, 28.8, 28.4, 27.7, 27.7, 24.8, 22.4.

**IR (cm$^{-1}$):** 3357, 3202, 2927, 2856, 1634, 1585, 1460, 1420, 1354, 1322, 1290, 1264, 1191, 1173, 1132, 1095, 1084, 1027, 977, 854, 836.

**HRMS (m/z):** $[M+Na]^+$ calculated for $C_{28}H_{36}O_6Na^+$, 491.2404; found, 491.2384.

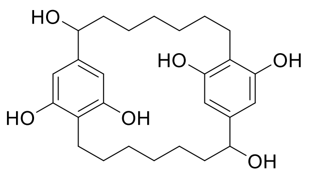


Palladium on barium sulphate (5 % Pd basis, 1.0 mg, 10 wt %) was added to a solution of **8** (10 mg, 0.0227 mmol) in acetone (5 mL). A $H_2$ atmosphere was applied and the reaction stirred at room temperature overnight. The mixture was filtered through a small pad of Celite and the filtrate evaporated. Purification by preparative HPLC (20–80 % B) afforded **9** (6.9 mg, 0.016 mmol, 68 %) as a white solid.

**$^{1}$H NMR (500 MHz, $(CD_3)_2SO$):** δ 8.72 (s, 4H), 8.71 (s, 4H), 6.11 (br s, 8H), 4.84 (d, *J* = 3.3 Hz, 2H), 4.82 (d, *J* = 3.4 Hz, 2H), 4.27 (app. qn, *J* = 3.7 Hz, 2H), 4.18 (app. qn, *J* = 3.8 Hz, 2H), 2.49-2.43 (m, 8H), 1.60–1.46 (m, 4H), 1.46–1.27 (m, 12H), 1.19-1.09 (m, 2H), 1.09-0.68 (m, 20H), 0.63–0.52 (m, 2H).

**$^{13}$C NMR (126 MHz, $(CD_3)_2SO$):** δ 155.5, 143.5, 143.4, 112.6, 112.4, 103.9, 72.9, 72.3, 39.4, 39.1, 30.7, 28.9, 28.8, 28.7, 26.0, 25.2, 22.0.

**IR (cm$^{-1}$):** 3314, 2930, 2855, 1624, 1598, 1434, 1366, 1290, 1210, 1146, 1101, 1084, 1021, 1013, 993, 841.

**HRMS (m/z):** $[M+Na]^+$ calculated for $C_{26}H_{36}O_6Na^+$, 467.2404; found, 467.2406.

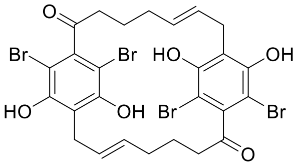


Pyridinium hydrobromide perbromide (polymer bound, ~2 mmol/g resin, 55 mg, 0.11 mmol) was added to a solution of **7b** (20 mg, 0.046 mmol) in ethanol (5 mL). The reaction was stirred at room temperature overnight. Then the mixture was filtered through a small pad of Celite and the filtrate evaporated. Purification by preparative HPLC (40–90 % B) afforded **10** (8.0 mg, 0.011 mmol, 23 %) as a yellow solid.

**$^1$H NMR (500 MHz, $(CD_3)_2SO$):** δ 9.13 (s, 4H), 5.42 (app. dt, $J$ = 15.1, 6.7 Hz, 2H), 5.34 (app. dt, $J$ = 15.1, 6.5 Hz, 2H), 3.36 (d, $J$ = 6.5 Hz, 4H), 2.53–2.48 (obsc. m, 4H), 2.07–2.01 (m, 4H), 1.67–1.61 (m, 4H).

**$^{13}$C NMR (126 MHz, $(CD_3)_2SO$):** δ 203.1, 151.5, 140.7, 129.9, 127.8, 118.4, 97.1, 40.6, 30.4, 27.8, 20.9.

**IR (cm$^{-1}$):** 3369, 2931, 1704, 1394, 1372, 1351, 1315, 1216, 1145, 1110, 1043, 1021, 989, 969, 822.

**HRMS (m/z):** $[M+H]^+$ calculated for $C_{26}H_{25}O_6Br_4^+$, 748.8385; found, 748.8382.

## Biochemical Materials

### Strains used in this work

| Strain | Description | Reference |
|---|---|---|
| *S. aureus* (Newman) | Methicillin-susceptible clinical isolate | Duthie *et al.*[2] |
| *S. aureus* (MRSA 15) | Methicillin-resistant clinical isolate | Richardson *et a.l*[3] |
| *E. faecalis* (Portland) | Vancomycin-susceptible clinical isolate | Andrewes *et al.*[4] |
| *P. aeruginosa* (PA01) | Wild-type | Stover *et al.*[5] |
| *S. marcescens* (Sma12) | Wild-type (Pig$^{-}$) clinical isolate. | Harris *et al*.[6] |

All media and reagents (analytical or molecular biology grade) were purchased from commercial vendors and used as received, and in accordance with the manufacturer's instructions where applicable.

LB refers to Luria Bertani Broth (Lennox); LBA refers to Luria Bertani Broth (Lennox) supplemented with 1.5 % agar. Likewise, MHB refers to Müller-Hinton Broth. All media was sterilised prior to use. MHB was adjusted to final cation concentrations of 20 mg $mL^{-1}$ $Ca^{2+}$ and 10 mg/mL $Mg^{2+}$.

## Biochemical Methods

### Bacteriology

Bacteria were grown to single colonies at 37 °C on LBA plates struck from -80 °C glycerol stocks. MHB was inoculated with 4–5 robust colonies and grown as a planktonic culture at 37 °C overnight. This was subcultured into LB (1:100) and grown as a planktonic culture at 37 °C to mid-log phase (~2 hours) as determined by $OD_{600}$. This culture was diluted as required (typically ~$1\times10^8$ cfu $mL^{-1}$) and used within 30 minutes.

### Determination of Minimum Inhibitory Concentrations (MIC)

We used the broth microdilution method reported by Hancock *et al.*[7] with the following adaptations: compounds were added as DMSO solutions not exceeding 1 vol % of the bacterial suspension (i.e. 1.5 μL in a total volume of 150 μL), and the final inoculum was increased to ~$1\times10^8$ cfu $mL^{-1}$. MICs for routine antibiotics (ciprofloxacin and gentamycin sulfate) were within the published range when tested against *S. aureus* and MRSA.

MICs were unaffected by the use of MHB unadjusted for cation content, shaking or shaking speed, or inoculum ($5\times10^6$ to $1\times10^9$ cfu $mL^{-1}$). Experiments were conducted in technical and biological triplicate. Growth inhibition was assessed visually after 20 hours and corroborated by $OD_{600}$ using an EZ Read 400 microplate reader (Biochrom). DMSO was used as the negative control. This method was also used to investigate the effect of pH on growth inhibition of compound **10**.

### Determination of Minimum Bactericidal Concentration (MBC)

At the end of the MIC assay, 100 μL from each well was diluted into PBS ($10^{-4}\rightarrow10^{-6}$); 100 μL aliquots were spread onto LBA plates. Viability was determined as cfu $mL^{-1}$ after 24 hours at 37 °C. The MBC was determined as the lowest concentration of **10** at which viability was 1000 times lower than the negative (DMSO) control.

### Killing Curves

*S. aureus* was grown as described above and diluted to $1\times10^8$ cfu $mL^{-1}$. This culture was divided into 1 mL aliquots and treated with compound **10** (0–200 μM final concentration). These were incubated at 37 °C with shaking (100 rpm). Samples were withdrawn over 8 hours, diluted as appropriate into PBS and spread onto LBA plates. Viability was determined

as cfu mL$^{-1}$ after incubation at 37 °C for 24 hours. Killing curves for *S. aureus* and MRSA were normalised to the negative (DMSO) control.

### Microscopy

*S. aureus* was grown as described above and diluted to 1×10$^{8}$ cfu mL$^{-1}$. This culture was divided into 1 mL aliquots and treated with compound **10** (0–200 μM final concentration). These were incubated at 37 °C with shaking (100 rpm). Samples were withdrawn over 4 hours and studied under 100× magnification.

### Epifluorescence Microscopy

Bacterial viability was estimated using a LIVE/DEAD BacLight Bacterial Viability Kit (Thermo Fisher) in accordance with the manufacturer's instructions.

### Determination of Proton Motive Force

*S. aureus* was grown as described above and diluted to 1×10$^{8}$ cfu mL$^{-1}$.

## References


[1] L. Leonard, B. Lygo, G. Proctor, *Advanced Practical Organic Chemistry*, Blackie Academic And Professional, Glasgow, **1995**.

[2] E. S. Duthie, L. L. Lorenz, *J. Gen. Microbiol.* **1952**, *6*, 95–107.

[3] J. F. Richardson, S. Reith, *J. Hosp. Infect.* **1993**, *25*, 45–52.

[4] F. W. Andrewes, T. J. Horder, *Lancet* **1906**, *168*, 708–713.

[5] C. K. Stover, X. Q. Pham, A. L. Erwin, S. D. Mizoguchi, P. Warrener, M. J. Hickey, F. S. L. Brinkman, W. O. Hufnagle, D. J. Kowalik, M. Lagrou, et al., *Nature* **2000**, *406*, 959–964.

[6] A. K. P. Harris, N. R. Williamson, H. Slater, A. Cox, S. Abbasi, I. Foulds, H. T. Simonsen, F. J. Leeper, G. P. C. Salmond, *Microbiology* **2004**, *150*, 3547–3560.

[7] I. Wiegand, K. Hilpert, R. E. W. Hancock, *Nat. Protoc.* **2008**, *3*, 163–175.
